# Global health: Global health diplomacy

**DOI:** 10.7189/jogh.10.020354

**Published:** 2020-12

**Authors:** Shinu Kuriakose

**Affiliations:** School of Health Professions, New York Institute of Technology, New York, Old Westbury, New York, USA

The issues inherent in the study of global health such as poverty, maternal and child morbidity and mortality, disease prevention, vaccinations, and the paucity of food and water do play an outsize role while framing foreign policy and diplomacy. These concepts can hamper or increase trade between countries, allow citizens of the developing world to be more economically sufficient, enjoy a middle-class lifestyle, decrease the intensity, frequency of diseases, and enhance screening of pathologies in a timely manner. A combination of global diplomacy, utilizing both private and public sector assistance, implemented in a robust manner can certainly help mitigate the issues faced by populations all over the world. Under the auspices of the World Health Organization (WHO), significant strides have been made to minimize the impact these global health conditions can cause countries and their citizens. “Global health diplomacy” (GHD) has been coined to describe the processes by which state and non-state actors engage to position health issues more prominently in foreign policy decision-making. Their ability to do so is important to advancing international cooperation in health [1].

The Oslo Declaration in 2010, signed by the foreign ministers of seven countries, tried to portray GHD in a framework where all diplomatic policy decisions must be taken in the context of how any proposed policy will affect health care in the region and important consideration must be given to the economic consequence of health care reform. There was a particular emphasis, by WHO, on collective collaboration among various countries and regions in recognition of the fact that a single deadly disease in one isolated area could turn out to be a pandemic in a very short period if measures were not in place to halt this spread. One of the basic tenets of GHD is that economic progress was not exclusive of good health care policies and procedures; rather, sound policies could enhance medical care all over the world, thus providing protection on a global scale.

## GENERAL AGREEMENT OF TRADE IN SERVICES (GATS)

GATS was conceived during the Uruguay round of trade negotiations from 1986-1993 and subsequently implemented in 1995; a concept which tried to recognize the importance of uniform trade rules on an international basis thus ensuring the sanctity of this process and helping it thrive; additionally, an underlying concept of this agreement was that this was a forum and opportunity for richer nations to help less developed ones. WHO perceives the importance of GATS in these two domains: “(a) ensuring increased transparency and predictability of relevant rules and regulations, and (b) promoting progressive liberalization through successive rounds of negotiations” [2]. This treaty emphasized the importance of the service sector and offered protection in the same way as the General Agreement on Tariffs and Trade (GATT) did for merchandise and commodities. All the members of the World Trade Organization (WTO) were also signatories to this agreement although there could be a temporary respite from certain regulations based on specific country requirements [3]. Although the goal of GATS is to remove trade barriers and create a conducive environment for trade, there are provisions for members to decide how fast they would like to take to deregulate certain service industries and set up individual benchmarks to do so.

Another advantage of the GATS agenda is the Most Favored Nation policy, which prohibits discrimination or withholding of services between countries, and tries to level the trade field; albeit, some exclusions, which are implemented in specific instances [4]. One of the criticisms of GATS is the fear by some entities that the government is ceding responsibility, and hence power over its citizens, by allowing business interests to operate freely without too many legal entanglements; a process, which they fear, will trump citizen's rights in favor of faceless business interests [5]. Furthermore, there is concern that if there are opposing viewpoints on a deal, a closed-door GATS disputes panel will solve these conflicts rather than rely on a country's judiciary system; a process which some feel will likely lead to favorable business decisions rather than humanistic concerns and be more prone to corruption.

## INTELLECTUAL GLOBAL HEALTH POLICY

Although there continues to be a debate on how to accurately debate GHD, there seems to be a consensus that this is an issue of global importance. There continues to be a robust debate on issues, which may be divergent in terms of economics vs purely health-related goals. Some examples include: promoting the trade of alcohol and nicotine over international borders although this is detrimental to health even though economically crucial to some countries, ensuring intellectual property rights and patents are protected for pharmaceutical drugs and vaccines while it can be price prohibitive in certain regions and using the guise of vaccinations to infiltrate terrorist or other organizations which may reduce faith in these public health programs [6]. “Intellectual property (IP) refers to creations of the mind, such as inventions; literary and artistic works; designs; and symbols, names, and images used in commerce” [7].

It has been estimated that out of 5000 to 10 000 experimental compounds studies, typically only one will gain Food and Drug Administration (FDA) approval; a process that lasts more than a decade and costs between 2-3 billion dollars [8]. A company would be hesitant to spend all these financial and labor resources on research and development to manufacture a drug only to see it chemically copied by shady companies in non-regulated nations. Furthermore, liability issues might also be involved if these for-profit companies are blamed for their medications (side or adverse effects) if not dosed in the appropriate fashion or indication. On the other hand, crucial medications might be held back from folks who cannot afford these high-cost medicines, which usually are not covered by medical insurance in the developing world. Thus, this ethical, legal, and financial quandary must take the GHP policy before formulating a final solution.

## VACCINATION/VIRUSES GLOBAL HEALTH POLICY

A major concern recently has been the emergence of viruses and other organisms, such as Ebola, which may initially emerge regionally but has the potential of exponentially expanding and becoming a pandemic. If countries are reluctant to have the WHO or the Centers for Disease Control (CDC) track these cases or point of origin in these countries, this could prevent a rational treatment and epidemiological study of these diseases. Thus, diplomatic channels may have to work overtime to encourage countries to change their worldview and recognize that specific decisions can have disastrous consequences. Furthermore, nations, which for religious, financial, or other nationalistic reasons, who withhold certain vaccinations from their citizens can put the whole world at risk due to the ease of transportation and travel in current times. An example of this would be the encouragement we receive by our health care providers to get the influenza vaccine because if enough folks get this vaccine, this “herd immunity” will also protect the immunocompromised and help prevent the spread of disease [9]. It is imperative that regions with known illnesses and spread of pathogens, due to insufficient medical protocols, be willing to engage and learn from more advanced countries with the reciprocity which would entail that their cooperation would help them receive treatments and vaccines at free or subsidized costs for their own citizens; an example of efficient global health policy.

**Figure:**
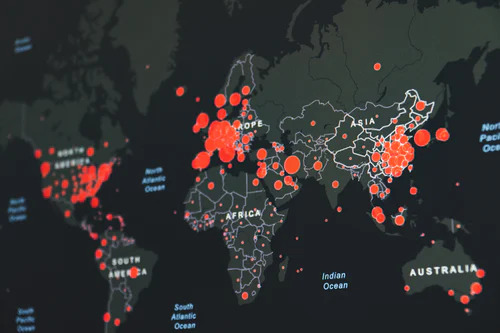
Photo: Score: https://unsplash.com/s/photos/global-health.

The world is in an unprecedented time during the COVID-19 pandemic with rates of illness and deaths climbing each day. Currently, there have been close to 8.5 million cases of COVID illness in the World with over 450 000 deaths [10]. This virus has caused an emotional, psychological, economic, sociological, and political challenge that the world has never faced before. It has given rise to “global health nationalism” aka “vaccine nationalism” [11], a phenomenon whereby countries place priorities to ensure that their citizens have access to vaccines, ventilators, medications to combat the COVID-19 virus prior to sharing it with the rest of the world. This nationalistic attitude when taken in isolation has led to political conflicts around the world spilling into trade wars and other rhetoric spewed by national leaders. Political leaders of countries are forced to explain to their citizens that valuable personal protective equipment (PPE’s) and other relevant resources are being withheld by their nations in case of a virus calamity affecting their country. This has led to charges of hoarding by nations where the virus has run rampant in the population leading to increased mortality and morbidity. In effect, countries are being prosecuted in the worldwide social media as monopolists who are not really part of the world community due to their nationalistic health care bent. The World Health Organization (WHO) has been shunned by a specific country due to a perception that China was being favored in terms of COVID preparation and lack of blame for not having enough safeguards to prevent COVID-19 spread. There still is a lack of fundamental global policy on the best mechanism for countries to share resources and move forward in a matter of benefitting all. Added to address “see the rise of ‘global health nationalism’ over who would get the ventilators, drugs, or vaccines first.”

## ENVIRONMENTAL GLOBAL HEALTH POLICY

Environmental health encompasses both chemical, biological, and physical factors in an environment, which can affect health [12]. In a bid to develop their nations to western standards, some developing countries are putting environmental concerns on the backburner and burning carbon fuels and other toxic gases, which can lead to greenhouse effects and other negative environmental situations. It is imperative that a robust effort must be taken not only by international organizations focused on climate change but also on countries that need to provide resources and technological know-how to nations that need vast amounts of natural resources to fuel their growing needs. This helps all involved, as there are less toxic gasses spewed with a lesser likelihood of polluting our planet. Not only would this decrease health care costs all over the world, but also this would lead to higher production by healthier workers which would benefit everyone; a win-win scenario indeed!

## CONCLUSION

Global Health Diplomacy encompasses a range of both abstract and specific actions, which both developed and developing countries can undertake, with the goal of helping all the citizens of the world. The right balance must be struck between both sound economical profits and a healthy global populace. The implementation of GATS in 1995 has been an impressive start to tackle this problem but one must make sure that all recommendations, which are part of this policy, be followed through with thorough oversight from global regulatory agencies. As the linking of the world makes everything, including diseases, seem less far away, it is imperative that rules be laid down and stringently followed in terms of vaccine distribution, screening of pathogens, disbursement in an ethical manner of legal drugs which cause harm (alcohol, nicotine) and timely policies to arrest disease in their tracks.

Countries across the world are currently facing a new reality, one which does not take into account socioeconomic status, race, gender, geographical location, developmental index, gross domestic product, level of the economy, the strength of the military, and type of government [13]. The Covid-19 virus should serve as a wake-up call to various stakeholders, particularly countries, in investing more resources in basic hygiene practices (handwashing, etc.), prevention of communicable diseases by instituting stringent protocols, transparency with not only their citizens but also the whole world when there is a disease outbreak, monitoring the food and water safety mechanisms, and encouraging their citizens to embrace a new reality. A robust global diplomatic policy, spearheaded by international organizations such as the United Nations, must take into account issues such as global health trade deficits, intellectual patent rights over medications and other medical devices, environmental safety recommendations, and a mechanism whereby resources would be shared in case of a global health emergency.

It is my view that although we may depend on governmental organizations initially to take the lead, they are beset with their own inherent social and economic problems and are answerable to their own citizens without paying too much heed to what is going on beyond their borders. Thus, it is prudent and crucial that non-governmental organizations under the auspices of agencies such as the United Nations, World Health Organization, and World Trade Organization take the initiative and implement programs, which will help encourage global health diplomacy with minimal disparities in regions. The Covid-19 pandemic creating havoc across the world had literally put 95% of the citizens of the world under “house arrest.” [14]. As technology progresses, humankind will be faced with an increasing number of health care maladies such as this (ironically, us being a victim of our own progress due to the fast spread of viruses with the world becoming increasingly interconnected” and all stakeholders will be forced to go back to the drawing board to draw up new plans for the likely unforeseen dangers ahead. This, of course, is easier said than done but the option of not going forward with this will be harmful to all involved and the recognition of this must lie in educating all consumers of this small planet of ours.
